# Ultrasound-based radiomics nomogram for predicting HER2-low expression breast cancer

**DOI:** 10.3389/fonc.2024.1438923

**Published:** 2024-09-18

**Authors:** Xueling Zhang, Shaoyou Wu, Xiao Zu, Xiaojing Li, Qing Zhang, Yongzhen Ren, Xiaoqin Qian, Shan Tong, Hongbo Li

**Affiliations:** ^1^ Department of Ultrasound Medicine, Affiliated Hospital of Nanjing University of Chinese Medicine, Nanjing, China; ^2^ Department of Ultrasound Medicine, Jiangsu University Affiliated People’s Hospital, Zhenjiang, China; ^3^ Materdicine Lab, School of Life Sciences, Shanghai University, Shanghai, China; ^4^ Department of Radiology, The Second Affiliated Hospital of Soochow University, Suzhou, China; ^5^ Department of Ultrasound Medicine, People’s Hospital of Longhua, Shenzhen, China

**Keywords:** breast cancer, HER2-low, radiomics, molecular subtype, ultrasound

## Abstract

**Purpose:**

Accurate preoperative identification of Human epidermal growth factor receptor 2 (HER2) low expression breast cancer (BC) is critical for clinical decision-making. Our aim was to use machine learning methods to develop and validate an ultrasound-based radiomics nomogram for predicting HER2-low expression in BC.

**Methods:**

In this retrospective study, 222 patients (108 HER2-0 expression and 114 HER2-low expression) with BC were included. The enrolled patients were randomly divided into a training cohort and a test cohort with a ratio of 8:2. The tumor region of interest was manually delineated from ultrasound image, and radiomics features were subsequently extracted. The features underwent dimension reduction using the least absolute shrinkage and selection operator (LASSO) algorithm, and rad-score were calculated. Five machine learning algorithms were applied for training, and the algorithm demonstrating the best performance was selected to construct a radiomics (USR) model. Clinical risk factors were integrated with rad-score to construct the prediction model, and a nomogram was plotted. The performance of the nomogram was assessed using receiver operating characteristic curve and decision curve analysis.

**Results:**

A total of 480 radiomics features were extracted, out of which 11 were screened out. The majority of the extracted features were wavelet features. Subsequently, the USR model was established, and rad-scores were computed. The nomogram, incorporating rad-score, tumor shape, border, and microcalcification, achieved the best performance in both the training cohort (AUC 0.89; 95%CI 0.836-0.936) and the test cohort (AUC 0.84; 95%CI 0.722-0.958), outperforming both the USR model and clinical model. The calibration curves showed satisfactory consistency, and DCA confirmed the clinical utility of the nomogram.

**Conclusion:**

The nomogram model based on ultrasound radiomics exhibited high prediction value for HER2-low BC.

## 1 Introduction

Breast cancer (BC) is the most frequent malignant disease among women globally, often associated with a high fatality rate (1). The human epidermal growth factor receptor 2 (HER2) emerges as a pivotal transmembrane tyrosine kinase receptor, regulating cellular proliferation and signal transduction. HER2, as a well-established biomarker of BC, often exhibits protein overexpression and gene amplification, which frequently correlates with an aggressive phenotype (2). According to the latest ASCO/CAP guidelines (3), HER2 status in BC is evaluated using immunohistochemistry (IHC) and/or fluorescence *in situ* hybridization (ISH). The HER2 status is now classified into three levels: HER2-0 (IHC score 0), HER2-low (IHC score 1 or 2/ISH negative), and HER2-positive (IHC score 3+ or 2/ISH positive). Previously, both HER2-0 expression and HER2-low expression BC were classified as HER2-negative BC. In reality, low levels of HER2 expression were observed in approximately two-thirds of hormone receptor (HR)-positive BC cases and in one-third of triple-negative BC (TNBC) cases (4). With the advancement of antibody-drug conjugates (ADCs) such as T-DXd and SYD985, the prognosis of HER2-low BC has improved.

Unlike BC with HER2-0 expression, HER2-low BC exhibits a unique incidence of somatic mutation, with higher rates of PIK3CA mutations and lower rates of TP53 mutations (5). Additionally, HER2-low BC typically presents with larger diameters, higher histological grading, and more involved lymph nodes. It has also been shown that the pCR rate is significantly decreased after neoadjuvant chemotherapy (6). Therefore, HER2-low expression is more likely to be an independent subtype, accounting for half of all BCs (7). It presents unique signaling pathways, molecular alterations, distinct biological features, and clinical outcomes (8). Hence, early detection of HER2-low expression is essential for clinical therapy planning.

At present, the preoperative diagnosis of the HER2-low expression primarily relies on the analysis of specimens obtained from core needle biopsy using IHC/ISH technique (9). However, due to the limited biopsy specimen size and the highly instability of HER2 low expressing during tumour development (10), samples may not fully capture all of the tumor’s properties. This highlights the need for the development of a non-invasive, precise, secure, and repeatable approach for predicting HER2-low expression.

Radiomics, as a high-throughput technology, offers a brand-new opportunity for HER2-low expression research. It is a hybrid field of computer science and medical imaging that aims to measure aspects that cannot be seen with the human eye (11). To strengthen the objectivity of tumor heterogeneity representation, radiation oncology makes use of more complete data mining, prediction, and analysis (12). Studies have reported that radiomics can effectively differentiate between benign and malignant breast lesions (13). Moreover, It also demonstrated excellent performance on molecular subtyping, lymph node metastasis prediction and neoadjuvant chemotherapy response prediction (14). Radiomics offers the advantages of safety and repeatability, enabling measurement of tumor heterogeneity at any time, unlike conventional biopsy techniques.

Ultrasound is widely recommended for preoperative assessment of BC patients. The rapid development of ultrasound-based radiomics (USR) technology has further spurred BC research (15). Given the scarcity of radiomics studies focused on HER2-low BC, our objective is to develop and validate a decision support tool.This tool will integrate clinical risk factors and USR to predict the HER2-low subtype in BC patients.

## 2 Materials and methods

### 2.1 Research population

**Figure 1:**
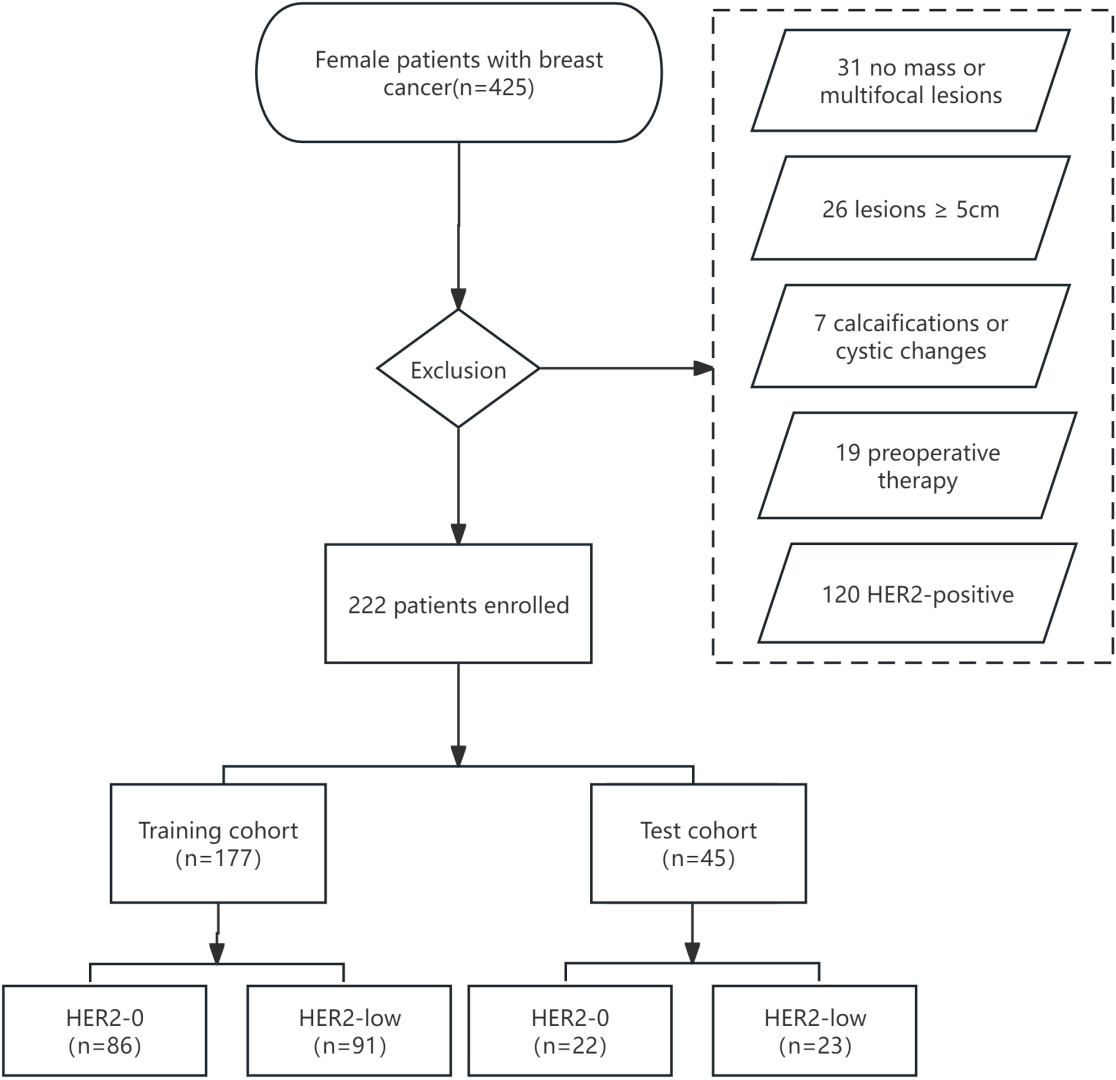
The flowchart of patient selection.

This retrospective analysis was approved by our institutional review board, and the requirement for informed consent was waived. We retrospectively reviewed 425 patients with histologically diagnosed BC between January 2019 and December 2021. The inclusion criteria were as follows: (і) female BC patients histologically confirmed by surgery or biopsy with complete clinical and pathological data; (ii) preoperative US performed within 1 week with complete imaging data. The exclusion criteria were as follows: (і) no-mass or multifocal lesions; (ii) lesions with calcifications or cystic changes that might significantly affect pixel values; (iii) lesions too large(≥5 cm) to be included in a single plane; (iv) patients who underwent preoperative therapy (neoadjuvant chemotherapy, biopsy before ultrasound); (v) HER2-positive (IHC score 3+ or 2+/FISH positive). **Figure 1** depicts the recruitment pathway.

### 2.2 Clinicopathological characteristics

**Figure 2:**
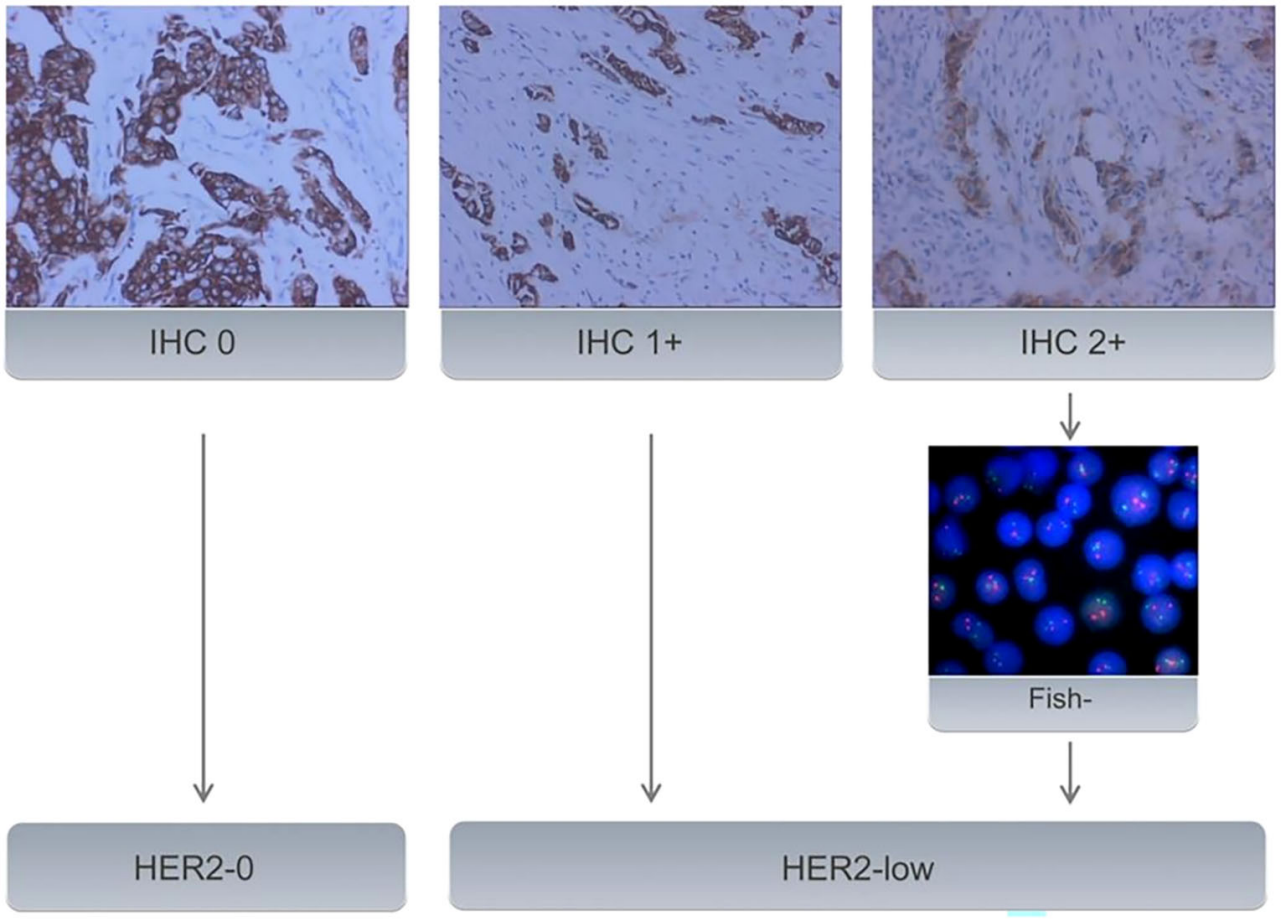
All samples with BC underwent IHC to evaluate HER2 status. Samples with an IHC score of 2+ underwent further evaluation using FISH. Samples with an IHC score of 0 were classified as HER2-0. IHC 1+ or FISH-negative results (no amplification) on IHC 2+ tumors were classified as HER2-low expression.

Baseline clinical data was obtained from medical records. The original pathological reports were retrieved to investigate pathology type, estrogen receptor(ER), progesterone receptor (PR), HER2, and Ki67 status. ER/PR status was marked positive if IHC staining in at least 1% of tumor nuclei; otherwise, it was marked as negative. The Ki67 level was categorized as low expression (<14%) and high expression(≥ 14%) (16). HER2-0 expression was defined as no membrane staining (IHC score 0), while HER2-low expression was defined as weak and incomplete membrane immunostaining with no HER2 gene amplification detected by FISH (IHC score 1 or 2+/FISH negative) (**Figure 2**).

### 2.3 Image acquisition and tumor segmentation

Preoperative US were conducted using a Philips Epiq5 (Philips, The Netherlands) or a GE LOGIQ E20 (GE Healthcare, USA) equipped with L12-5 or ML6-15 linear array transducers. The US examinations were completed by skilled sonographers with at least 7- year experience. All patients were scanned in a supine position with both breasts fully exposed. The biggest slice of each lesion was obtained, and US characteristics were noted according to the Breast Imaging Reporting and Data System (BI-RADS) (17).

Sonographer 1 (with more than 7 years of experience) selected the maximum cross-sectional plane of each breast lesion and manually segmented a Region of Interest (ROI) along the tumor margin using ITK-SNAP software (version 3.8). The segmention was confirmed by sonographer 2 (with more than 10 years of experience). Both sonographers were blinded to US and clinicopathological results.

### 2.4 Feature extraction and selection

Features based on US images were extracted using PyRadiomics (Version 2.2.0). These features encompassed first-order statistics, two-dimensional (2D) shape descriptors, texture classes, and higher-order features, including gray-level co-occurrence matrix (GLCM), gray-level run length matrix (GLRLM), gray-level size zone matrix (GLSZM), gray-level dependence matrix (GLDM), neighbourhood gray-tone dependency matrix (NGTDM), and filter-based features. Only features with the interclass correlation coefficient (ICC) greater than 0.75 were retained for further analysis (18).

Berore feature selection, Z-score standardization was applied for data preprocessing. This adjustment aimed to set the mean value to zero, eliminate disparities in value scales, and mitigate the impact of outliers. It is formulated as Z = (X - μ)/σ, where Z stands for transformed value, μ stands for mean, and σ stands for standard deviation. Afterward, the enrolled patients were randomly allocated into a training cohort and a test cohort in an 8:2 ratio. Feature selection was conducted within the training cohort. The candidate USR features significantly associated with HER2-low expression were filtered out by an independent samples t-test. The least absolute shrinkage and selection operator (LASSO) regression was then employed to reduce the dimensionality of the high-dimensional data and eliminates redundant features by utilizing L1 regularization to reduce the regression coefficients of unnecessary features to zero. To prevent overfitting, the optimal regularization parameter lambda was determined via a 5-fold cross-validation process. In the 5-fold cross-validation, the training dataset was divided into five subsets. For each iteration, four of these subsets were designated for model training, while the remaining one served as the validation set. After completing all five iterations, the most relevant USR features with non-zero coefficients associated with HER2-low expression were screened out.

### 2.5 Model construction and performance validation

**Figure 3:**
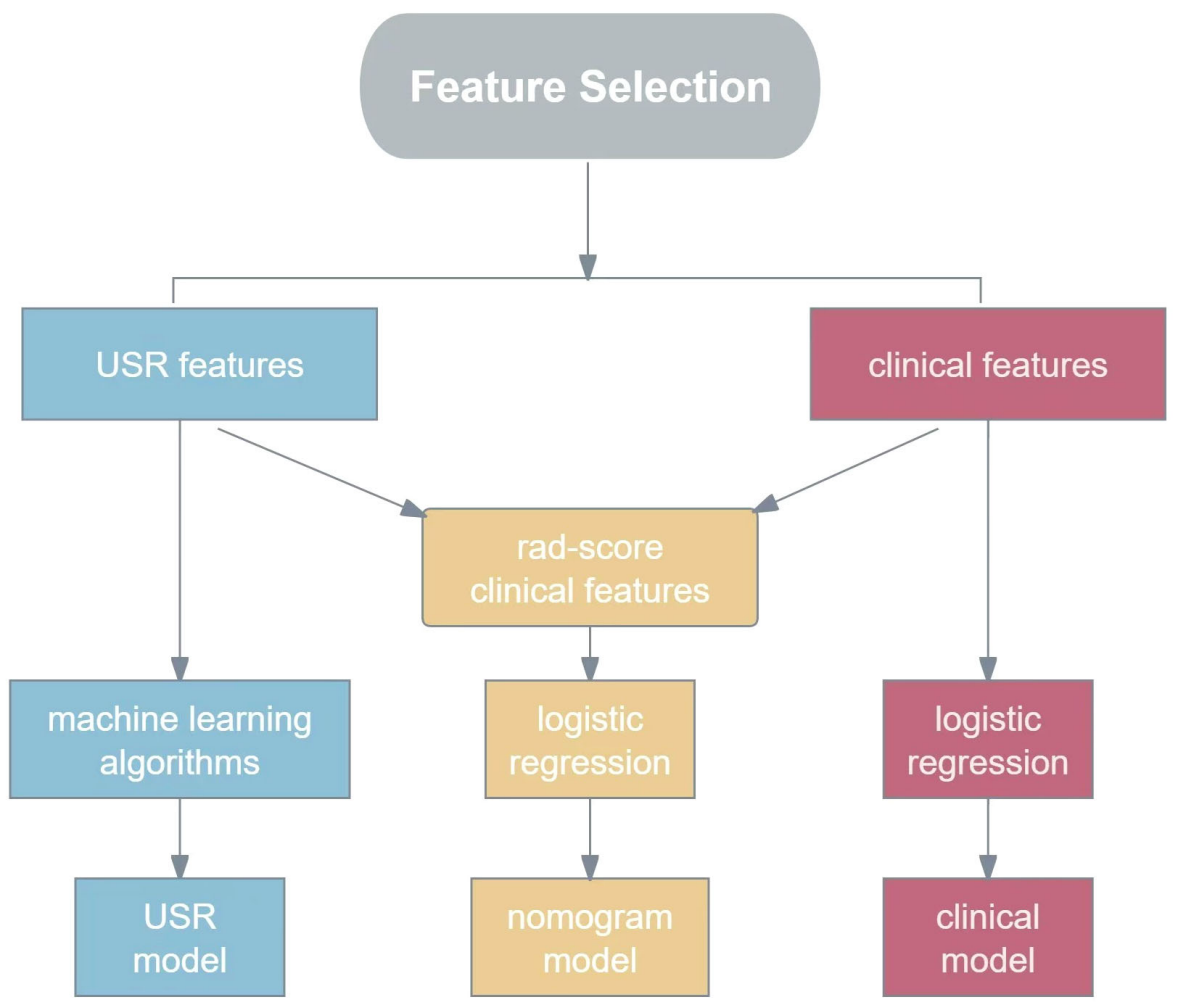
The flowchart for constructing USR model, clinical model and nomogram model. USR, ultrasound-based radiomics.

On the basis of these USR features, the following five machine learning algorithms were used for model training: Support Vector Classification with Linear Kernel (SVC-L), Support Vector Classification with Radial Basis Function Kernel (SVC-RBF), Extreme Gradient Boosting (XGB), Logistic Regression (LR), and Gaussian Naive Bayes (GNB).The performance of each algorithm was assessed, and the best-performing one was chosen as the USR model. These USR features were converted to radiomics score (rad-score) through a calculation involving the multiplication of radiomics eigenvalues by their respective LASSO coefficients, followed by summation. To establish a clinical model, multivariable logistic regression analyses were performed to identify clinical risk factors associated with HER2-low expression, with statistical significance defined as a p-value<0.05. Subsequently, a nomogram was developed combining rad-score with clinical risk factors(R language, package “rms”). The construction workflow is shown in **Figure 3**.

The model was trained on the training dataset, and its performance was evaluated on the test dataset. Receiver operating characteristic (ROC) curves were plotted for each model separately, and the area under the curve (AUC) was calculated. Furthermore, accuracy, sensitivity, and specificity were calculated to evaluate the discrimination ability of the models. Calibration curves were plotted to evaluate the performance of the nomogram, with the fitness assessed by the Hosmer-Lemeshow (H-L) test (*p*>0.05). Decision curve analysis (DCA) was used to determine the clinical practicability of the prediction models by quantifying the net benefits.

### 2.6 Statistical analysis

Statistical analysis was performed by R software (version 3.6.1, https://www.r-project.org). Quantitative data were presented as mean ± standard deviation(x ± s). Quantitative data conforming to the normal distribution were compared by independent sample t-test, whereas the nonparametric Mann-Whitney U test was applied for grade data. Pearson Chi-square test and Fisher exact test were used to compare categorical data. A two-sided *p*-value<0.05 was considered statistically significant. ROC curves and DCA curve were plotted using Python (version 3.1) and AUCs 95% confidence intervals (95% CI) were computed. Goodness of fit was assessed by the H-L test. Pairwise comparisons of the ROC curves were performed using the DeLong test.

## 3 Results

### 3.1 Baseline clinicopathologicl and ultrasonic characteristics

Eventually, a total of 222 cases were enrolled (mean age, 56.01 ± 12.66 years; mean tumor size, 2.19 ± 1.09cm). Among them, 108 cases (48.6%) showed HER2-0 expression and 114 cases (51.4%) exhibited HER2-low expression. All cases were randomized into two cohorts:training cohort (n = 177) and test cohort (n = 45). No significant differences were observed between the training and test cohorts (p>0.05, **Supplementary Table S1**). Compared to the HER2-0 group, characteristics such as shape, microcalcification, ER, and PR differed statistically significant in the HER2-low group. In particular, the HER2-low group had greater rates of HR positivity (ER+ 79.8% vs. 66.6%; PR+ 75.4% vs. 60.1%) (*p*<0.05; **Table 1**).

**Table 1:** Comparison of baseline characteristics of patients between the HER2-0 and HER2-low BC by univariate analysis.

Characteristics	HER2-0 (n=108)	HER2-low (n=114)	*p*-value
Age (year, mean ± SD)	56.34 ± 12.59	55.70 ± 12.76	0.707
Size (cm, mean ± SD)	2.28 ± 1.21	2.12 ± 0.96	0.280
Position			0.064
outer upper quadrant	51	61	
outer lower quadrant	10	16	
upper inner quadrant	16	22	
lower inner quadrant	16	9	
central	15	6	
Border			0.252
clear	45	39	
fuzzy	63	75	
Shape			0.011*
regular	16	33	
irregular	92	81	
Aspect ratio			0.310
<1	47	42	
≥1	61	72	
Microcalcification			<.001*
without	85	64	
within	23	50	
Internal blood flow grade			0.134*
adler0	35	33	
adler1	38	31	
adler2	21	26	
adler3	14	24	
ER			0.027*
negative	36	23	
positive	72	91	
PR			0.015*
negative	43	28	
positive	65	86	
Ki67			0.051
low expression	25	40	
high expression	83	74	
Histologic type			0.118
DCIS	6	8	
non-special invasive	87	92	
infiltrative specific	10	14	
rare	5	0	

*p<0.05. Alder blood flow grade, 0 no flow, 1 punctate blood flow signal, 2 short-rod like blood flow signal, 3 abundant blood flow signal. DCIS, ductal carcinoma in situ.

### 3.2 Radiomics feature selection and USR model construction

480 USR features were extracted from each original US image with good agreement of the feature extraction (ICC>0.75), including 19 firstorder features, 10 shape features, 75 texture features, and 376 higher-order composite features. Following an independent sample t-test, 110 features demonstrating statistically significant differences from HER2-low expression were further screened out. The optimal parameter (λ = 0.0308) for Lasso regression was identified by 5-fold cross-validation, resulting in the best model fit. After dimensionality reduction, 11 USR features with non-zero coefficients were retained finally. The majority of these features were wavelet-transformed (9/11), while the remaining two were LoG-transformed features (**Table 2**). Based on this foundation, five machine learning algorithms were introduced to compare the performance. Furthermore, a comparison of the performance in terms of accuracy, specificity, and sensitivity of these five algorithms is presented in **Table 3**. All algorithms have passed the validation with none exhibited overfitting. In the test cohort, the performance of all algorithms decreased because the test cohort comprised image data that were unknown previously. This decrease in performance of the test cohort reflects the generalization ability of the models. Among them,the SVC-RBF algorithm gave the highest AUC value (AUC=0.78). In addition, we noticed a decrease in sensitivity and a slight increase in specificity (60.9% and 77.3%, respectively). The overall performance of SVC-RBF is more balanced across all algorithms and was selected as the USR model.

**Table 2:** Eleven retained non-zero coefficients features after dimensionality reduction.

Filter	Category	Feature Name	Mean Value		*p*-value	Feature Coefficient
			HER2-0	HER2-low		
LoG	gldm	LargeDependenceLow GrayLevelEmphasis	0.8538	0.5669	0.001	-0.041862
LoG	glrlm	LowGrayLevel RunEmphasis	0.3596	0.2866	0.011	-0.004805
wavelet	firstorder	InterquartileRange	8.7533	11.4926	<0.01	0.025894
wavelet	firstorder	Range	32.804	42.177	0.002	0.064012
wavelet	glcm	InverseVariance	0.4265	0.3363	0.025	-0.038853
wavelet	firstorder	Minimum	-51.7193	-19.5611	<0.01	0.062032
wavelet	glcm	Correlation	0.5932	0.4198	0.019	-0.021779
wavelet	glcm	JointAverage	5.6366	4.1785	0.011	-0.048355
wavelet	gldm	GrayLevel NonUniformity	4.3905	3.6691	0.01	-0.006254
wavelet	glrlm	GrayLevel NonUniformity	3.4017	2.8856	0.035	-0.026538
wavelet	glrlm	GrayLevelNon UniformityNormalized	0.1531	0.1256	0.016	-0.014143

**Table 3:** Performance of the five machine learning classifiers in training and test cohort.

Classifier	Cohort	AUC	95%CI	ACC(%)	SEN(%)	SPE(%)
SVC-L	Training	0.78	0.707-0.844	69.5	69.2	69.7
	Test	0.77	0.628-0.906	60	43.5	77.3
SVC-RBF	Training	0.86	0.800-0.912	78.5	81.3	75.5
	Test	0.78	0.650-0.919	68.9	60.9	77.3
XGB	Training	0.83	0.772-0.892	75.1	83.5	66.3
	Test	0.71	0.564-0.865	60	56.5	63.6
LR	Training	0.77	0.700-0.838	51.4	100	0
	Test	0.76	0.615-0.898	57.8	43.5	72.7
GNB	Training	0.76	0.692-0.832	67.8	81.3	53.4
	Test	0.69	0.532-0.843	66.7	65.2	68.7

**Figure 4:**
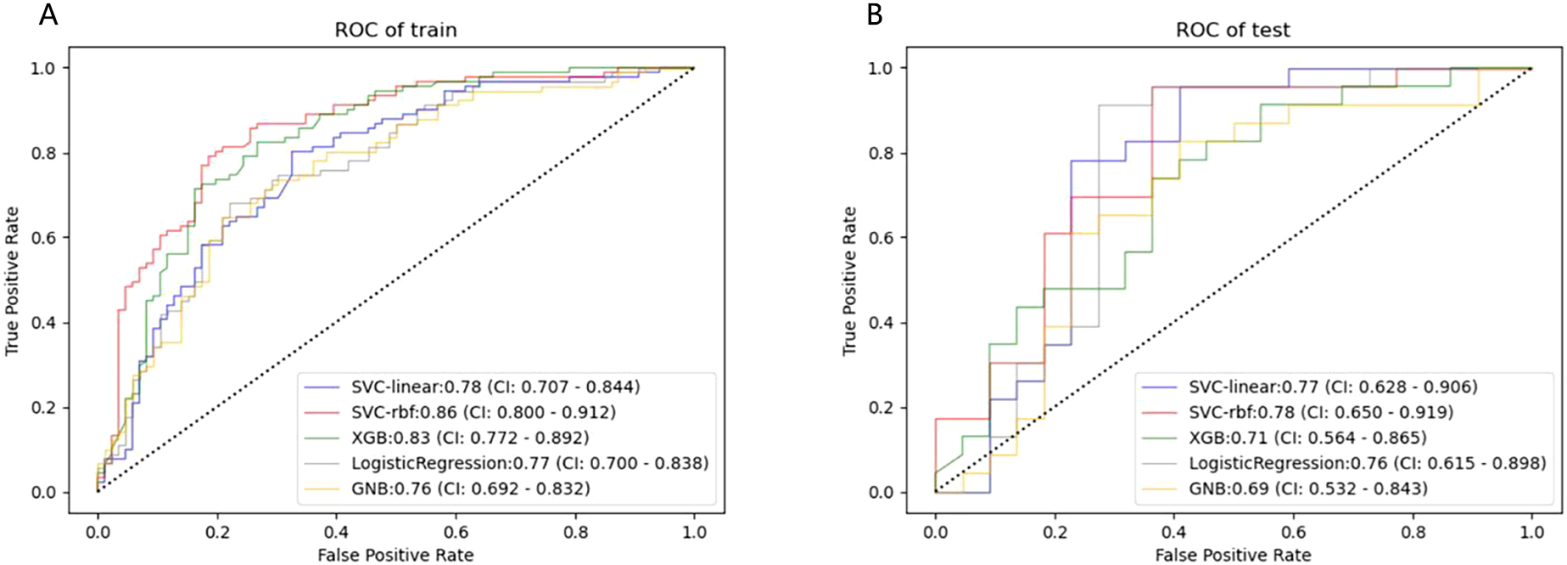
ROC curves of the five machine learning classifiers predicting HER2-low expression in the training **(A)** and test cohort **(B)**.

The SVC-RBF performed best out of all algorithms (training AUC, 0.86; test AUC, 0.78). **Figure 4** presents a comparison of the ROC curves for the five algorithms. These 11 USR features were considered highly associated with HER2-low expression, and output as rad-score ( Radscore=coefficient 1×feature 1+coefficient 2×feature 2…+ constant term) (19).

**Figure 5:**
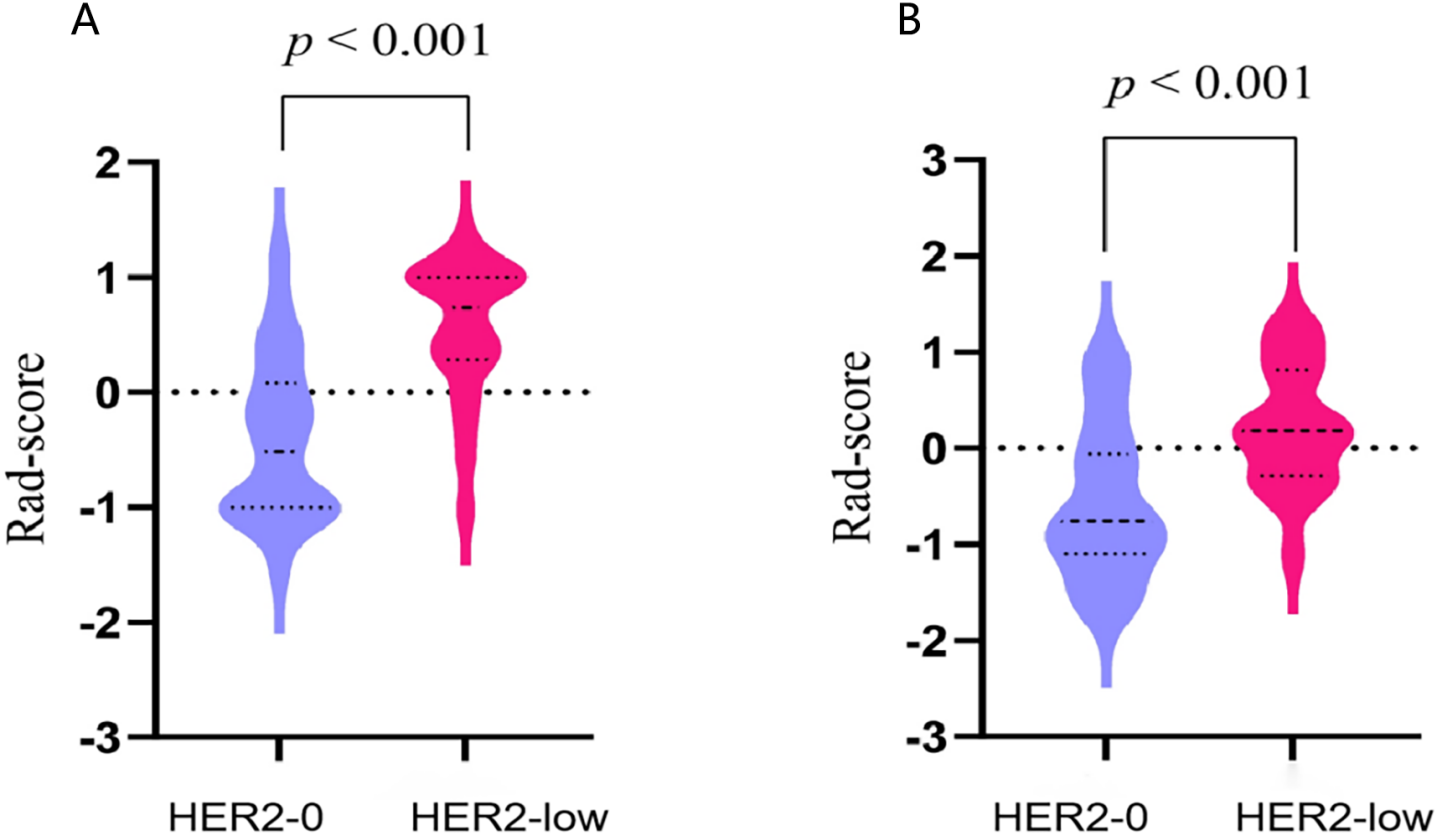
The violin plots illustrating the distribution of rad-scores between the HER2-0 and HER2-low groups in the training **(A)** and test cohort **(B)**.

The rad-score of each patient was calculated. The median rad-score of the HER2-low group was found to be significantly higher than that of the HER2-0 group (*p*<0.001; **Figure 5**).

### 3.3 Clinical model and nomogram model construction

Multivariate analysis revealed several baseline characteristics that were statistically significant between HER2-low expression and HER2-0 expression, including tumor border, shape, and microcalcification (*p*<0.05; **Table 4**). Hence, these characteristics were incorporated as clinical risk factors in the construction of the clinical model. Nevertheless, its performance fell below expectations, with training and test AUC values of 0.61 and 0.58, respectively.

**Table 4:** Multivariate analysis for the classification of HER2-0 and HER2-low BC.

Characteristics	*p*-value	OR (95%CI)
Age	0.890	1.00 (0.98 - 1.03)
Size	0.486	0.89 (0.64 - 1.24)
Position	0.492	1.39 (0.55 - 3.54)
Border	0.020*	3.11 (1.19 - 8.12)
Shape	<.001*	0.15 (0.05 - 0.44)
Aspect ratio	0.967	1.01 (0.54 - 1.91)
Microcalcification	0.003*	2.70 (1.39 - 5.26)
Internal blood flow grade	0.994	1.00 (0.45 - 2.23)
ER	0.673	0.79 (0.27 - 2.34)
PR	0.367	1.58 (0.59 - 4.24)
Ki67	0.247	0.65 (0.31 - 1.35)
Histologic type	0.321	1.94 (0.52 - 7.19)

*p<0.05.

**Figure 6:**
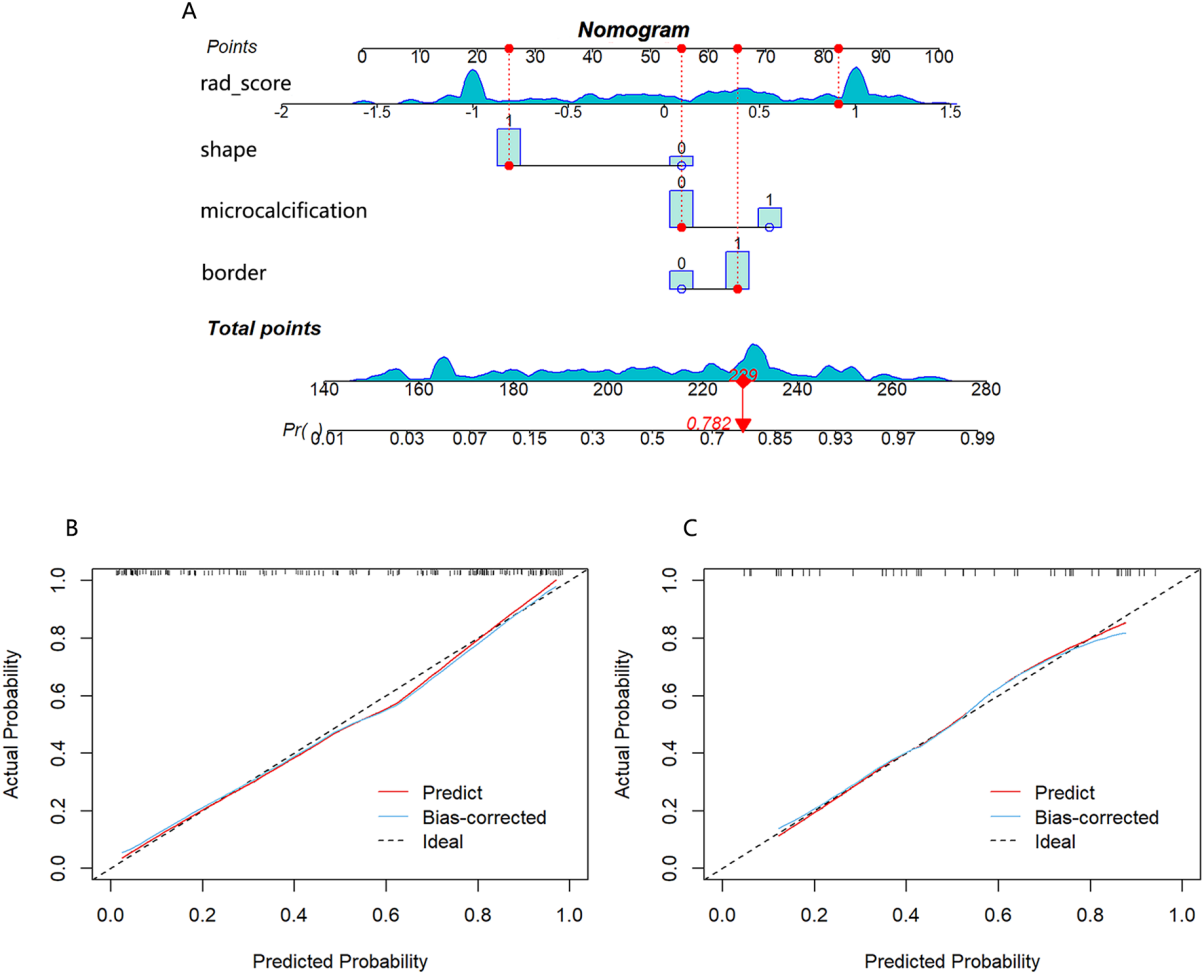
**(A)** Nomogram developed with shape, border, microcalcification, and rad-score. If a patient had a rad-score of 0.841, a mass with irregular shape, unclear border and no calcification, the general nomogram score was 229, with a corresponding probability of HER2-low expression is 0.782. Calibration curve of the nomogram in the training cohort **(B)** and test cohort **(C)**.

A nomogram combining the rad-score with clinical risk factors was constructed (**Figure 6A**). The nomogram model was established as follows: HER2-low Expression Prob $=\left(-1.739\times 10^{-6}\right)\times {\text{points}}^{3}+0.000392327\times {\text{points}}^{2}-0.015125275\times \text{points}+0.158781371$ . The nomogram model demonstrated a good distinguishing ability between HER2-0 expression and HER2-low expression, as evidenced by the greatest AUC values (training AUC, 0.89; test AUC, 0.84). The calibration curves were close to the 45-degree lines, and the H-L test showed there was no significant difference between the observed value and expected value (training *p*=0.671, test *p*=0.541), signifying strong agreement and high calibration precision of the model (**Figures 6B, C**).

### 3.4 Model performance assessment

**Figure 7:**
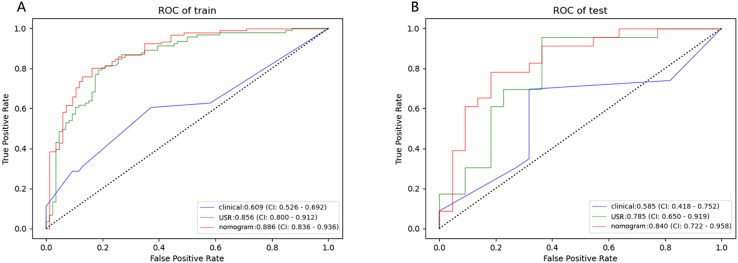
Performance of the clinical model, USR model, and nomogram model. ROC curves in training cohort **(A)** and test cohort **(B)**. USR, ultrasound-based radiomics.

**Figure 8:**
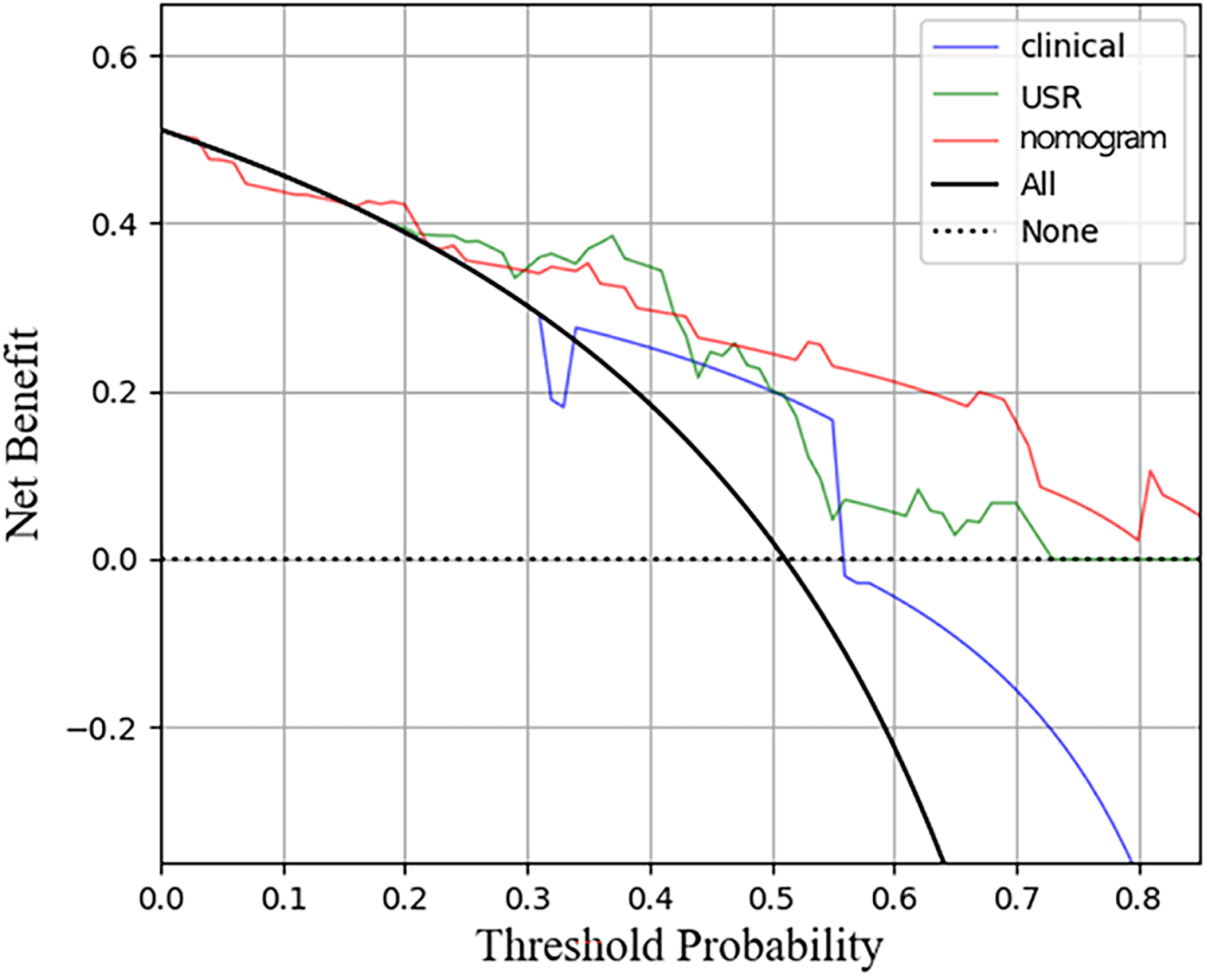
Decision curve analysis for the three models in test cohort. The horizontal axis represents measures the net benefit and the vertical axis measures the threshold probability. USR, ultrasound-based radiomics.

The nomogram model exhibited the highest performance (training AUC, 0.89; test AUC, 0.84), followed by the USR model (training AUC, 0.86; test AUC, 0.78), and then the clinical model (training AUC, 0.61; test AUC, 0.58) (**Table 5**). **Figure 7** displays the ROC curves of the three models. Statistical analysis using the Delong test revealed that the nomogram model showed a significantly higher AUC than the other models (*p*=0.043, *p*<0.001, respectively) (**Table 6**). **Figure 8** illustrates the DCA of these models. It demonstrated that within the threshold range of 0.29-0.42, the nomogram model provided a higher net benefit compared to the USR and clinical model.

**Table 5:** Performance of the three prediction models in training and test cohort.

Model	Cohort	AUC	95%CI	ACC(%)	SEN(%)	SPE(%)
Clinical	Training	0.61	0.526-0.692	68.9	69.5	68.2
	Test	0.58	0.418-0.752	61.6	60.4	62.8
USR	Training	0.86	0.800-0.912	78.5	81.3	75.5
	Test	0.78	0.650-0.919	68.9	60.9	77.3
Nomogram	Training	0.89	0.836-0.936	83.6	84.6	82.5
	Test	0.84	0.722-0.958	73.3	65.2	81.8

USR, ultrasound-based radiomics.

**Table 6:** AUC comparison of three prediction models by Delong test.

	z-value	*p*-value
USR model vs clinical model	4.815	<0.001*
USR model vs nomogram model	-2.021	0.043*
clinical model vs nomogram model	6.635	<0.001*

*p<0.05.

## 4 Discussion

In this retrospective study, we explored the feasibility of radiomics features extracted from ultrasound images as noninvasive predictors to differentiate between HER2-low expression and HER2-0 BC. Additionally, we developed a nomogram by integrating clinical factors with radiomics features for HER2-low BC prediction.

Traditionally, the assessment of HER2 status in BC has been binary, categorizing tumors as either negative or positive. However, advancements in genomic and molecular research advocate for a more precise ternary classification of HER2 expression status in clinical practice. HER2-low expression BC likes to be an unique subtype with distinct molecular, pathological, and clinical characteristics. Encouragingly, novel ADCs have provided targeted therapy opportunity for BC patients with HER2-low expression (20). Early and accurate diagnosis of HER2-low BC contributes to the improvement of prognosis.

The inherent instability of HER2 expression in BC, due to its high heterogeneity, presents challenges in accurately assessing tumor landscape through biopsies, particularly with limited sample sizes. A previous study reported that 18.5% of cases had post-operative pathology inconsistent with preoperative puncture results (21). Besides, the availability of FISH testing is constrained by local laboratory conditions, leading to inconsistencies in HER2 status interpretation across different centers (22). Hence, novel non-invasive approaches are needed to effectively evaluate HER2-low expression.

Ultrasound is a commonly used modality in the clinical assessment of BC. Our study identified that border, shape, and microcalcification as independent clinical risk factors. The clinical model was established based on these ultrasound features. We observed that the border and shape characteristics of HER2-0 BC are more relatively clear and regular compared to the HER2-low group, consistent with previous findings in TNBC patients (23). The molecular mechanisms of microcalcifications are not fully understood but may be linked to SPCA2-Orai1 (24). HER2 amplification can activate Orai channels, leading to calcium release inside the cell. Ultrasound lacks sensitivity in detecting microcalcifications, and the definition of tumor shape and borders is reliant on the subjective judgment of sonographers. This may explain the poor performance of our clinical model. Son et al. (25) similarly reported AUCs of merely 0.501, 0.680, and 0.665 for discriminating HER2-positive type, Luminal type, and TNBC based on the clinical model. Thus, relying solely on clinical parameters may not be adequate for accurately predicting BC molecular subtypes.

It is not surprising, given the considerable biological intricacies and documented variation in HER2 gene expression and somatic mutations among HER2-low BC cases. HER2 is a crucial negative prognostic factor in BC, facilitating the activation of signaling cascades responsible for cell proliferation, migration, and survival (26). HER2 presented a heterogeneous amplification, with instability and high degree of variations even in different intrinsic subtypes (27). The heterogeneity likely maps as subtle morphological, structural, and functional distinctions which is imperceptible for human eye, but could be quantitatively evaluated by radiomics (28), providing additional deeper insights into the tumor.

The USR model developed in this study, which includes 11 features utilizing wavelet or LoG filter, demonstrated a significant improvement in performance. Among these features, wavelet features were found to have the greatest weight (9 out of 11), making them the most pivotal aspects in this radiomics analysis. Taking the wavelet feature with the largest weight coefficient as an example(wavelet-H_firstorder_Range), it represents the variation between maximum and minimum of gray-level values within the image. With the modification of tumor heterogeneity, the grey level distributions will likewise be altered (29). The wavelet transform is a flexible and robust tool that utilizes different basic wavelet functions to decompose signals (30). It enables the extraction of multiscale features in both time and frequency domains,thereby capturing finer details in images (31). Zhou et al. (32) observed that peritumoral textures in wavelet-transformed images are characterized by high-frequency signals, whereas intratumoral textures can be characterized by low-frequency signals. It enhances the information content in low-frequency signals (33). Jiang et al. (34) reported that wavelet-transformed radiomics outperformed original first-order and texture radiomics (AUC: 0.921 vs. 0.880). Moreover, wavelet features contribute to predict lymph node metastasis, vascular invasion and oncogene amplification (35, 36).

In this study, the 11 USR features were converted into rad-score. It was observed that the HER2-low group displayed a higher median score in comparison to the HER2-0 group. The increased rad-score reflects a greater variability in gray levels and more complex textures within the ROI (37). Xu et al. also observed a higher rad-score in the TNBC group compared to the non-TNBC group (38). Tumors with high heterogeneity typically manifest complex local textures, varying gray levels, and irregularly borders (39). Consequently, our study provides evidence of the heightened degree of heterogeneity in HER2-low BC.

Recent studies have emphasized the emerging trend of integrating radiomics with clinical factors, with the expectation that this integration may offer enhanced value (40). A previous research combined the rad-score with age and BIRADS criteria in a nomogram, resulting in superior discrimination between TNBC and fibroadenoma (AUC 0.986 and 0.977, respectively). Likewise, the nomogram model developed by Hu et al. (41), demonstrated commendable performance in microvascular invasion prediction (validation AUC of 0.731). In this study, we constructed a nomogram to leverage the advantages of integrating clinical risk factors with the rad-score. To the best of our knowledge, this is the first application of ultrasonography-based radiomics for predicting HER2-low expression. The nomogram model demonstrated superior performance compared to those achieved by the clinical model or the USR model individually (AUC: training 0.89; test 0.84). The calibration curves indicated satisfactory consistent and accurate predictions by the nomogram, while DCA analysis demonstrated a higher net benefit associated with its clinical use.

There are several limitations to this study that must be acknowledged: (і) Small sample size: our study’s small sample size raises the possibility of selection bias. (ii) Limited clinical features: our study included a relatively small number of clinical features. As research on HER2-low BC is still in its early stages, future efforts may benefit from adding more data from labs and molecular detection. Therefore, the potential of clinical characteristics may not have been fully utilized in our current investigation. (iii) Monomodal ultrasound imaging: we solely utilized two-dimensional grayscale ultrasound images, excluding multimodal techniques like Doppler ultrasound, ultrasound elastography, and contrast-enhanced ultrasound. Multi-modal ultrasound technology may bring more complementary information.

## 5 Conclusions

Ultrasound-based radiomics has a good predictive ability for HER2-low expression in BC. The nomogram model integrates clinical factors and rad-score, yielding the highest predictive performance.This non-invasive methodology shows potential for early assessment of HER2-low expression, which is crucial for clinicians in guiding treatment decisions for BC patients.

## Data Availability

The raw data supporting the conclusions of this article will be made available by the authors, without undue reservation.
