# Supplementary material for: Ultrasound-based radiomics nomogram for predicting HER2-low expression breast cancer
Source: Front Oncol. 2024 Sep 18;14:1438923. doi: 10.3389/fonc.2024.1438923 (PMC11445231; doi:10.3389/fonc.2024.1438923)
Supplement: Supplementary file 1 [file Table1.docx]

**Table S1. Comparison of Baseline Characteristics of Patients Between the Training and Test Cohort**

| Characteristics | Training cohort(n=177) | Test cohort(n=45) | *p*-value |
| --- | --- | --- | --- |
| Age (year, mean ± SD) | 56.25±12.50 | 55.07±13.37 | 0.575 |
| Size (cm, mean ± SD) | 2.20±1.12 | 2.18±0.99 | 0.886 |
| Position |  |  | 0.564 |
| outer upper quadrant | 89 | 23 |  |
| outer lower quadrant | 22 | 4 |  |
| upper inner quadrant | 28 | 10 |  |
| lower inner quadrant | 19 | 6 |  |
| central | 19 | 2 |  |
| Border |  |  | 0.993 |
| clear | 67 | 17 |  |
| fuzzy | 110 | 28 |  |
| Shape |  |  | 0.405 |
| regular | 37 | 12 |  |
| irregular | 140 | 33 |  |
| Aspect ratio |  |  | 0.723 |
| ＜1 | 72 | 17 |  |
| ≥1 | 105 | 28 |  |
| Microcalcification |  |  | 0.669 |
| without | 120 | 29 |  |
| within | 57 | 16 |  |
| Internal blood flow grade |  |  | 0.312 |
| alder0 | 59 | 9 |  |
| alder1 | 51 | 18 |  |
| alder2 | 37 | 10 |  |
| alder3 | 30 | 8 |  |
| ER |  |  | 0.717 |
| negative | 48 | 11 |  |
| positive | 129 | 34 |  |
| PR |  |  | 0.618 |
| negative | 58 | 13 |  |
| positive | 119 | 32 |  |
| HER2 |  |  | 0.971 |
| 0 | 86 | 22 |  |
| low | 91 | 23 |  |
| Ki67 |  |  | 0.300 |
| low expression | 49 | 16 |  |
| high expression | 128 | 29 |  |
| Histologic type |  |  | 0.074 |
| DCIS | 12 | 2 |  |
| non-special invasive | 139 | 40 |  |
| infiltrative specific | 23 | 1 |  |
| rare | 3 | 2 |  |

HER2, Human epidermal growth factor receptor 2; ER, estrogen receptor; PR,progesterone receptor.
